# Unraveling the molecular mechanisms in severe Alzheimer’s disease based on transcriptomic data using next generation knowledge discovery methods

**DOI:** 10.12669/pjms.42.1.11019

**Published:** 2026-01

**Authors:** Hind A. Alkhatabi, Alaa G. Alahmadi, Muhammad Imran Naseer, Peter Natesan Pushparaj

**Affiliations:** 1 Dr. Hind A. Alkhatabi, Ph.D. Department of Biological Science, College of Science, University of Jeddah, Jeddah, Saudi Arabia; 2 Dr. Alaa G. Alahmadi, Ph.D. Department of Biological Science, College of Science, University of Jeddah, Jeddah, Saudi Arabia; 3 Prof. Muhammad Imran Naseer, Ph.D, Institute of Genomic Medicine Sciences, King Abdulaziz University, Jeddah-21589, Saudi Arabia; 4 Dr. Peter Natesan Pushparaj, Ph.D, Institute of Genomic Medicine Sciences, King Abdulaziz University, Jeddah-21589, Saudi Arabia

**Keywords:** Alzheimer’s disease, Dorsolateral prefrontal cortex, Gene set enrichment analysis, Ingenuity pathway analysis, Next generation knowledge discovery, Neuroinflammation, rRNA processing, RNA sequencing, WebGestalt

## Abstract

**Background & Objectives:**

Alzheimer’s disease (AD) is characterized by gradual cognitive decline. Here, we deciphered the molecular mechanisms using transcriptomic data derived from the dorsolateral prefrontal cortex (DLPFC) of patients with severe AD using next-generation knowledge discovery (NGKD) techniques.

**Methodology:**

RNA sequencing data from the Gene Expression Omnibus (GEO) database (GSE53697) derived from the DLPFC of individuals with severe AD and healthy controls, obtained originally from frozen brain tissues of individuals classified based on the Consortium to Establish a Registry for Alzheimer’s Disease (CERAD) criteria, and differentially expressed genes (DEGs) were identified by GEO2R analysis. The WEB-based GEne SeT AnaLysis Toolkit (WebGestalt) was used for overrepresentation analysis (ORA) using the Kyoto Encyclopedia of Genes and Genomes (KEGG) Pathway Database and Gene Set Enrichment Analysis (GSEA) using the KEGG, Reactome, and Wiki pathway databases. Ingenuity Pathway Analysis (IPA) software was used to decode the key canonical pathways and gene networks implicated in severe AD.

**Results:**

We identified 24,207 DEGs using P ≤0.05, and this list was further filtered with a fold change cut-off ±1.5 to derive 3103 genes. WebGestalt analysis showed that retrograde endocannabinoid signaling, motor proteins, oxidative phosphorylation, and ribosome biogenesis were downregulated, whereas pathways related to immune system activation, such as antigen processing and presentation and cytokine signaling, were enriched in patients with severe AD. IPA analysis showed significant downregulation of ribosomal RNA (rRNA) processing and enrichment of neuroinflammatory signaling pathways. Crucially, dysregulation of energy metabolism, protein synthesis, axonal transport, and immunological responses have been identified in the DLPFC of patients with severe AD.

**Conclusion:**

Using NGKD methods, we identified an array of molecular pathways implicated in neuroinflammation, dysregulation of energy metabolism, and mitochondrial damage in severe AD and their association with disease progression. Our findings add to the existing knowledge on the pathophysiology of severe AD and help in the development of more effective strategies for diagnosis, therapy, and prevention.

## INTRODUCTION

Alzheimer’s disease (AD) is characterized by the gradual accumulation of extracellular β-amyloid (Aβ) plaques and intracellular neurofibrillary tangles composed of tau protein in the brain.^1^,^2^ Patients therefore exhibit progressive cognitive decline, memory loss, and behavioral changes.^1^–^3^ Typical features of cognitive loss in patients are memory impairment, aphasia, apraxia, agnosia, and disorientation.^3^,^4^ In addition, patients with AD may experience epileptic seizures, which complicate the clinical management of this debilitating disease.^5^ AD remains the fifth-leading cause of death among Americans aged 65 and older.^3^,^5^ Approximately 6.9 million Americans aged ≥65 years suffer from AD-associated dementia in the United States of America (USA).^3^,^5^ It was predicted that by 2060, 13.8 million individuals will be affected by AD in the absence of proper therapeutics to prevent, ameliorate, or cure AD. Globally, over 55 million individuals live with dementia, and the number of people with AD will double every two decades, potentially reaching approximately 78 million by 2030 and 139 million by 2050. An estimated 60–80 % of dementia cases are linked with AD and the majority of individuals experiencing brain changes and associated abnormalities due to a variety of etiologies of dementia.^3^,^5^

Due to difficulties in evaluating individuals with cognitive impairment^6^, the exact frequency of epileptic seizures in patients with AD remains unknown. Studies have reported glial activation, synaptic dysfunction, and marked neuronal loss in patients with severe AD.^7^-^9^ Oxidative stress, neuroinflammation, protein misfolding, and mitochondrial dysfunction are some of the characteristics of severe AD.^10^,^11^ Devising therapeutic modalities and improving treatment options depends on the understanding of molecular signaling pathways implicated in advanced or severe AD.

Recent findings based on next-generation knowledge discovery (NGKD) strategies have significantly expanded our knowledge and provided cues for further exploration of complex cellular and molecular mechanisms, enabling the identification of unique molecular markers, gene expression signatures, and protein-protein interactions associated with severe AD.^3^,^12^-^14^ As the exact mechanisms underlying the control of severe AD remain unknown, we investigated the complex molecular pathways associated with severe AD using NGKD tools. The dorsolateral prefrontal cortex (DLPFC) is vital for cognitive processes and memory.^15^ It was observed that the DLPFC displays structural atrophy like reduced cortical thickness and functional abnormalities, including diminished neuroplasticity and altered connectivity and are associated with cognitive impairments. 15Herein, we decipher the RNA sequencing (RNA-seq) data from the DLPFC of patients with severe AD using NGKD techniques.^16^

## METHODOLOGY

**Fig.1:**
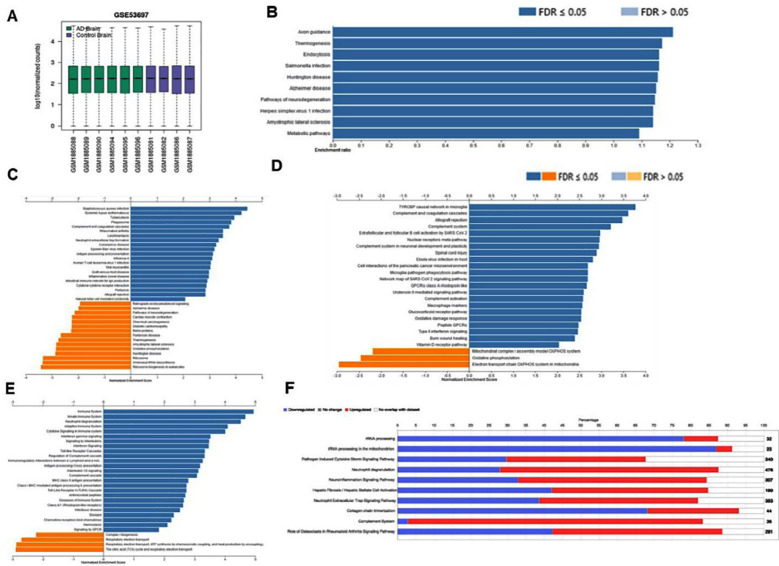
(A) RNA-seq data from the Gene Expression Omnibus (GEO) (accession no: GSE53697) were filtered, and only four controls and six severe AD samples were selected for further downstream analysis using next-generation knowledge discovery (NGKD) methods. **(B)** Overrepresentation analysis (ORA) of differentially expressed genes (DEGs) obtained from GEO2R analysis with a P-value ≤ 0.05, with Benjamini-Hochberg (BH) multiple testing correction based on the Kyoto Encyclopedia of Genes and Genomes (KEGG) pathway database. **(C)** Differentially regulated KEGG pathways, **(D)** Reactome pathways, **(E)** WikiPathways, and **(F)** canonical pathways based on Ingenuity Pathway Analysis (IPA) software core analysis in patients with severe AD.

We used RNA-seq data from the Gene Expression Omnibus (GEO) database (accession number: GSE53697) for this study. The dataset was deposited in GEO by Scheckel et al. (2016).^15^ Here, frozen brain tissues were obtained from the Mount Sinai Brain Bank of individuals classified based on the Consortium to Establish a Registry for Alzheimer’s Disease (CERAD) criteria.^15^The DLPFC tissue was further processed, and RNA was extracted using the TRIzol method, followed by RNA-seq using an Illumina HiSeq 2500 (Illumina Corporation, USA).^15^ Although the study by Scheckel et al.^15^ (2016) included eight control subjects (without tangles or plaque pathology) and nine patients with advanced or severe AD (CDR 4-5), matched for age and sex, with brief postmortem intervals (PMI), we filtered and selected only four controls and six advanced AD samples (Fig.1A) for further downstream analysis using NGKD methods, as previously described.^12^-^14^

### Ethical statement:

Our study did not require ethical approval because it did not involve human or animal subjects and used existing RNA sequencing (RNA-seq) datasets from GEO. It was conducted entirely in a virtual environment and thus poses no risks to living subjects; therefore, Institutional Review Board (IRB) approval is not required for this study.^3^,^13^,^14^

### GEO2R analysis:

The RNA-seq data from GSE53697 were analyzed with GEO2R using a robust multi-array average (RMA) algorithm to clean the background noise and ensure comparability between samples by reducing the technical variability and increasing the accuracy of downstream analyses using NGKD tools (access date: September 23, 2024). GEO2R was used to decipher differentially expressed genes (DEGs) between healthy controls and patients with severe AD using the statistical package “Linear Models for Microarray and RNA-Seq Data - limma” in R.^14^ For this purpose, linear models were fitted to the expression data for each gene by using empirical Bayes techniques to control for standard errors. Significant DEGs were selected using specific statistical thresholds such as P≤0.05 and the false discovery rate (FDR) using the Benjamini-Hochberg (BH) multiple testing correction method.

### WebGestalt analysis:

An overrepresentation analysis (ORA) of DEGs in advanced AD compared to the control was performed using the WEb-based GEne SeT AnaLysis Toolkit (WebGestalt) (wGSEA) and the Kyoto Encyclopedia of Genes and Genomes (KEGG) Pathway Database with a P-value threshold of ≤ 0.05.^17^ As previously reported^3^,^13^, DEGs were further filtered using a fold-change criterion of ± 1.5 and analyzed by Gene Set Enrichment Analysis (GSEA) using WebGestalt (access date: September 23, 2024). Pathway analysis was performed using KEGG, Reactome, and Wikipathway databases. We selected Homo sapiens as the species, the gene symbol as ID, the genome as reference, and a minimum of five and a maximum of 2000 IDs (enrichment criteria) in each category, using the BH method with a significance level of P ≤ 0.05 to calculate the FDR.

### Ingenuity pathway analysis:

Ingenuity Pathway Analysis (IPA) software (Qiagen, USA) was used to deduce the differentially regulated canonical pathways, upstream regulators, and non-directional gene networks in severe AD based on DEGs filtered with a cut-off of ± 1.5 and a P-value of less than 0.05. 13,18 Fisher’s exact test was used in IPA to calculate the Z-scores. Differentially regulated canonical pathways, upstream regulators, and undirected gene networks were visualized using IPA to further deduce AD-associated disease mechanisms.

## RESULTS

GEO2R analysis of the RNA sequencing data derived from the DLPFC region of the brains of patients with severe AD and the normal control regions (Fig.1A) provided 24207 DEGs based on a P-value cut-off of ≤ 0.05, with BH multiple testing correction for FDR. Furthermore, this list was filtered based on a fold change of ± 1.5 to obtain 3103 DEGs for downstream processing and knowledge discovery. We used the WebGestalt tool to initially analyze the 24207 DEGs based on the Over Representation Analysis (ORA) method to evaluate the impact of advanced AD on the molecular mechanisms based on the KEGG pathway database (Fig.1B) and found that the pathways of neurodegeneration, Huntington’s disease, endocytosis, axon guidance, amyotrophic lateral sclerosis (ALS), Salmonella Infection, and metabolic pathways were significantly enriched (P ≤ 0.05) based on FDR in patients with AD.

Next, we used the GSEA method in WebGestalt to evaluate the DEGs filtered based on fold change to obtain differentially regulated KEGG, Reactome, and WikiPathways in patients with AD. Based on this analysis, we found that KEGG pathways such as AD, aminoacyl t-RNA biosynthesis, oxidative phosphorylation, motor proteins, amyotrophic lateral sclerosis (ALS), neurodegeneration, ribosome biogenesis in eukaryotes, and retrograde endocannabinoid signaling were significantly downregulated (P ≤ 0.05) in the AD group compared to the control group. In contrast, the DEGs implicated in neutrophil extracellular trap formation, Epstein-Barr virus infection, Staphylococcus aureus infection, antigen processing, and presentation were significantly (P ≤ 0.05) enriched in the AD group than in the control group (Fig.1C).

Reactome pathways, such as ATP synthesis by chemiosmotic coupling, Complex I biogenesis, respiratory electron transport, heat production by uncoupling proteins, and the citric acid (TCA) cycle, were negatively enriched (P ≤ 0.05) in patients with AD compared with the control group. Conversely, the Reactome pathways, such as GPCR signaling, infectious disease, immune system, cytokine, and chemokine signaling, were positively (P ≤ 0.05) enriched in the DLPFC of patients with AD compared to that of the control group (Fig.1D).

The electron transport chain oxidative phosphorylation (OXPHOS) system in mitochondria and mitochondrial complex I assembly model OXPHOS system based on WiKiPathways were negatively enriched (P ≤ 0.05) in patients with AD compared to controls. In contrast, WikiPathways, such as the complement system in neuronal development and plasticity, oxidative damage response, Type-II interferon signaling, tyrosine protein tyrosine kinase-binding protein (TYROBP) causal network in microglia, complement and coagulation cascades, Ebola virus infection in the host, and microglial pathogen phagocytosis pathway, were positively (P ≤ 0.05) enriched in patients with AD compared to the control group (Fig.1E). The canonical pathways from the IPA core analysis revealed that ribosomal RNA (rRNA) processing, transfer RNA (tRNA) processing in the mitochondria, and collagen chain trimerization were downregulated (Z-score ≤ -2). In contrast, canonical pathways, such as the pathogen-induced cytokine storm signaling pathway, neutrophil degranulation, neuroinflammation signaling pathway, neutrophil extracellular trap signaling pathway, and complement system, were enriched (Z score ≥ 2) in patients with severe AD compared to the control group (Fig.1F).

**Fig.2:**
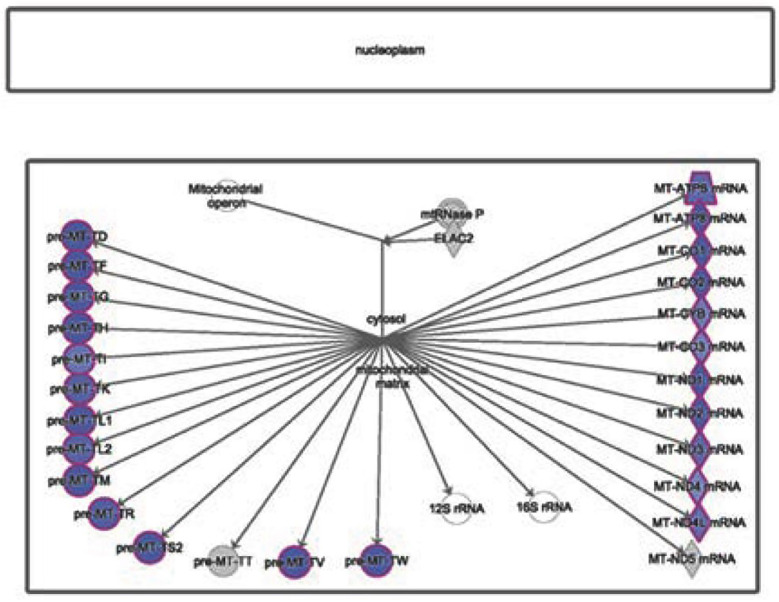
Ribosomal RNA (rRNA) RNA-processing pathway in AD. The downregulated genes involved in the rRNA processing canonical pathway were obtained from IPA software (based on negative Z-scores, highlighted in blue) in severe AD.

IPA analysis showed that genes encoding enzymes involved in rRNA processing, such as cytochrome c oxidase subunit I, cytochrome c oxidase subunit III, NADH dehydrogenase subunit 1, ATP synthase F0 subunit 8, NADH dehydrogenase subunit 2, NADH dehydrogenase subunit 3, ATP synthase F0 subunit 6, and cytochrome c oxidase subunit II (Fig.2), were negatively regulated in the severe AD group.

**Fig.3:**
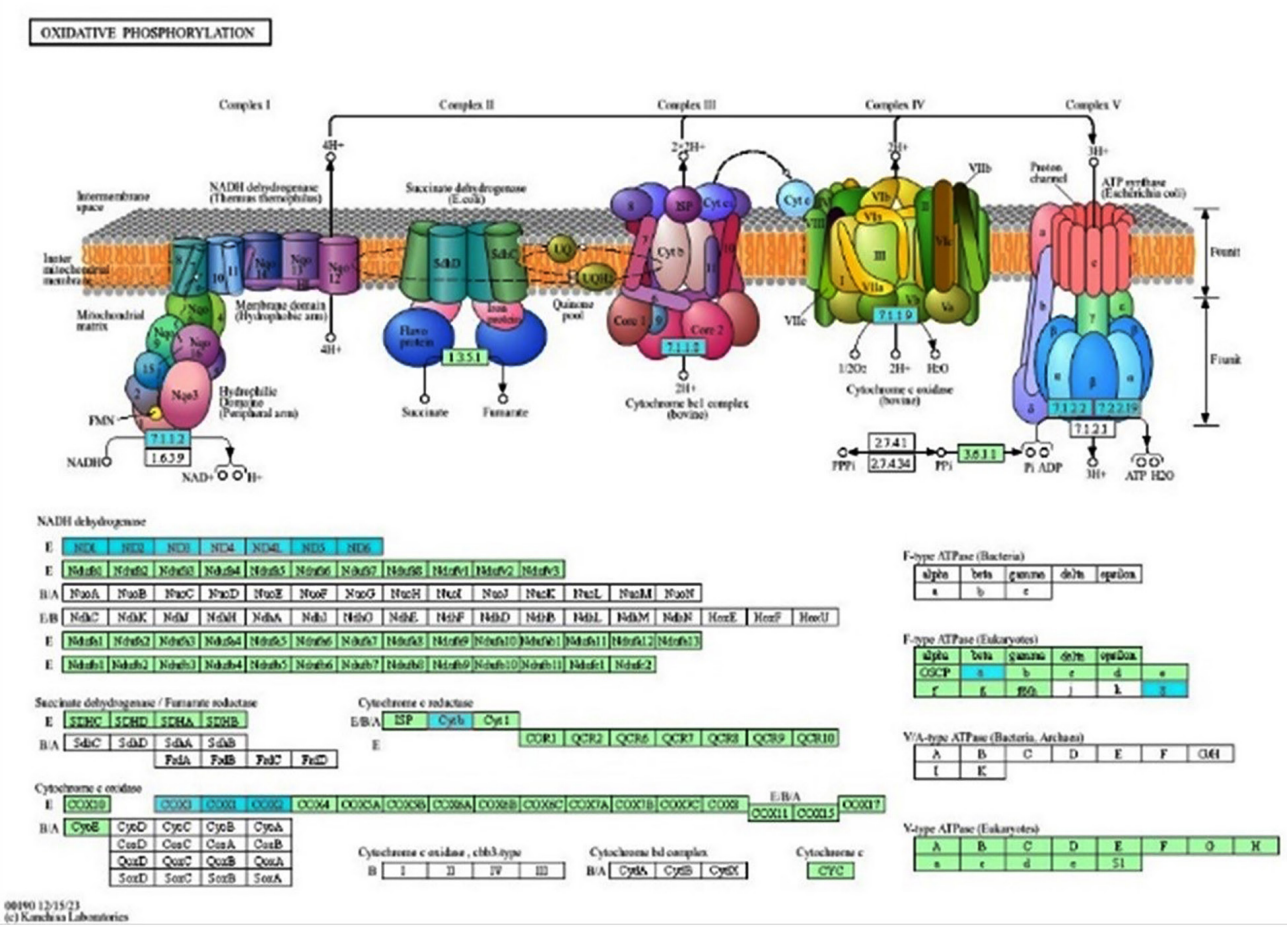
Oxidative phosphorylation pathway based on the KEGG database in severe AD. The downregulated genes involved in oxidative phosphorylation (highlighted in turquoise) in severe AD were identified using the GSEA method in WebGestalt.

The genes involved in oxidative phosphorylation (highlighted in turquoise), such as NADH dehydrogenase subunit 2, ATP synthase F0 subunit 8, NADH dehydrogenase subunit 6, ATPase H+/K+ transporting subunit alpha, cytochrome c oxidase subunit I, cytochrome c oxidase subunit II, ATP synthase F0 subunit 6, and NADH dehydrogenase subunit 4, based on KEGG pathway database were significantly downregulated (P ≤ 0.05) in patients with AD (Fig.3).

**Fig.4:**
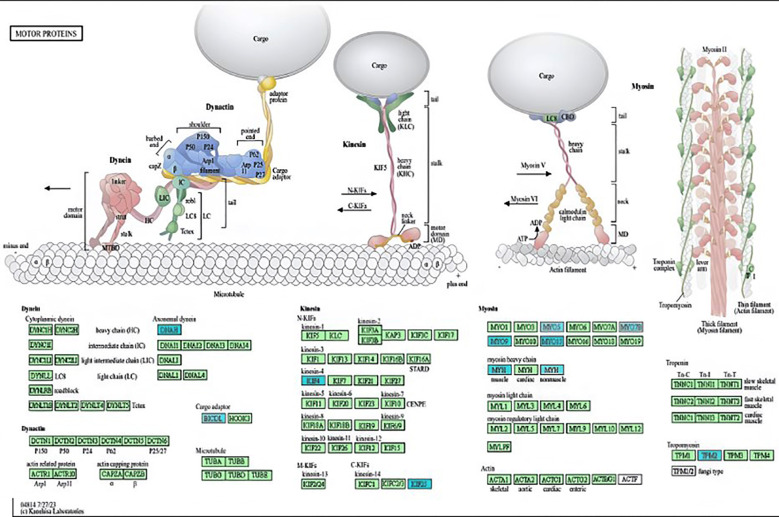
Motor protein pathways based on the KEGG database in patients with severe AD. Downregulated genes involved in the motor protein pathway (highlighted in turquoise) in severe AD were derived using the GSEA method in WebGestalt.

Moreover, KEGG pathway analysis showed that genes coding for motor proteins (highlighted in turquoise), such as dynein axonemal heavy chain 1, myosin heavy chain 3, kinesin family member 25, myosin IXB, kinesin family member 4 B, myosin XVB, tropomyosin 2, and BICD family members, such as cargo adaptor 1, dynein axonemal heavy chain 2, myosin heavy chain 7 B, myosin VIIB, myosin VC, and myosin heavy chain 14, were significantly (P ≤ 0.05) downregulated in AD patients (Fig.4).

Interestingly, an array of significantly downregulated genes (P ≤ 0.05) involved in neurodegeneration pathways (Table-I), including dynein axonemal heavy chain 1, NADH dehydrogenase subunit 6, mitogen-activated protein kinase 12, NADH dehydrogenase subunit 1, ATPase sarcoplasmic/endoplasmic NADH dehydrogenase subunit 3, cytochrome c oxidase subunit III, NADH dehydrogenase subunit 4, cytochrome c oxidase subunit I, reticulum Ca2+ transporting 1, cytochrome b, NADH dehydrogenase subunit 5, dynein axonemal heavy chain 2, and NADH dehydrogenase subunit 4 L, were also involved in AD signaling and OXPHOS in AD patients.

**Table-I:** List of differentially regulated genes implicated in various pathways involved in neurodegeneration in AD.

S. No.	Gene Name	Gene Symbol	Entrez Gene ID	Score
1	NADH dehydrogenase subunit 6	ND6	4541	-2.4743
2	NADH dehydrogenase subunit 2	ND2	4536	-1.9249
3	Cytochrome c oxidase subunit II	COX2	4513	-1.5505
4	NADH dehydrogenase subunit 1	ND1	4535	-1.5232
5	NADH dehydrogenase subunit 5	ND5	4540	-1.5209
6	Dynein axonemal heavy chain 1	DNAH1	25981	-1.5036
7	Dynein axonemal heavy chain 2	DNAH2	146754	-1.4354
8	Cytochrome c oxidase subunit I	COX1	4512	-1.3766
9	ATP synthase F0 subunit 8	ATP8	4509	-1.3395
10	ATPase sarcoplasmic/endoplasmic reticulum Ca2+ transporting 1	ATP2A1	487	-1.2187
11	NADH dehydrogenase subunit 3	ND3	4537	-1.163
12	ATP synthase F0 subunit 6	ATP6	4508	-1.1612
13	NADH dehydrogenase subunit 4L	ND4L	4539	-1.1259
14	Mitogen-activated protein kinase 12	MAPK12	6300	-1.0747
15	Cytochrome b	CYTB	4519	-1.0096
16	Cytochrome c oxidase subunit III	COX3	4514	-0.9737
17	NADH dehydrogenase subunit 4	ND4	4538	-0.9415

## DISCUSSION

AD is characterized by a slow loss of cognitive abilities, memory, and behavior that affects daily life and quality of life (QoL).^1^,^2^,^3^,^12^ In the present study, we observed marked changes in gene expression potentially regulating an array of molecular signaling pathways in the DLPFC of patients with severe AD compared to healthy controls. Here, the NGKD analysis of DEGs derived from patients with severe AD, based on both open-source tools such as WebGestalt and IPA, a commercial software, provided pivotal evidence of the differently regulated complex molecular landscape in severe AD.

Our NGKD analysis revealed the dysregulation of axon guidance, endocytosis, and various neurodegenerative processes in severe AD, as observed in previous studies. 3,18,19 The dysregulation of axon guidance leads to abnormal neuronal connectivity and impaired endocytosis which can significantly impair amyloid-beta clearance, thus increasing the accumulation of pathological aggregates in the AD brain.^20^–^22^ AD dysregulates retrograde endocannabinoid signaling, motor proteins, and oxidative phosphorylation. Synaptic plasticity, neuroprotection, and inflammation control are dependent on these factors. Disruption of this signaling pathway in AD can lead to progressive loss of neurons and cognitive impairment. 23 Cognitive decline in AD has been linked to dysfunction of the endocannabinoid system. Proper axonal transport is essential for maintaining ideal neuronal health and synaptic functions; hence, the dysregulation of motor proteins involved in axonal transport contributes to synaptic dysfunction and neuronal loss in AD.^24^,^25^ More importantly, the dysregulation of motor protein genes, as observed in our study in severe AD, can lead to the accumulation of harmful proteins and organelles, causing a slow loss of neurons in the brain. In addition, the NADH dehydrogenase subunits and cytochrome c oxidase subunits involved in the electron transport chain are downregulated in AD, which could significantly impair energy metabolism in the neurons of patients with AD. Mitochondrial dysfunction may substantially reduce ATP production, increase oxidative stress, and impair calcium homeostasis, thereby resulting in neuronal death in patients with AD, as previously described.^3^ Antigen processing and presentation, neutrophil extracellular trap (NET) formation, and cytokine signaling are upregulated in AD. As observed in our study, the immune activation in the brain could result in the release of pro-inflammatory mediators, increased oxidative stress, and neurotoxicity, further exacerbating neuronal damage or loss of neurons and resulting in progressive cognitive decline.^26^-^28^

The immune responses against pathogens such as Staphylococcus aureus and Epstein-Barr virus may further augment the chronic inflammatory state observed in AD brains, as we observed that the molecular pathways related to these microbes were positively enriched in the DLPFC of patients with severe AD.^29^ Ribosome biogenesis plays an important role in normal neuronal function^30^,^31^, and the accumulation of tau proteins and β-amyloid (Aβ) (1-42) causes progressive neuronal loss, the formation of neuronal fiber tangles (NFTs), and senile plaque (SP) deposition in neurons.^32^ Studies have demonstrated that pathological tau proteins are abnormally associated with ribosomes in AD brains, leading to impaired protein synthesis.^33^ This aberrant tau-ribosome interaction significantly reduces the production of nascent proteins that are important for synaptic plasticity and are critical for learning and memory. 30, 31, 34 Ribosomal dysfunction that leads to impaired translation has been associated with memory loss in tauopathies such as AD.^34^

Furthermore, studies have demonstrated that tau oligomerization induces translational stress response and inhibits protein synthesis. An array of ribosomal proteins was identified based on mass spectrometry that selectively bind to oligomeric tau (oTau), thereby affecting quality control mechanisms related to ribosomal biogenesis and RNA translation.^35^ These findings provides a mechanistic link between the presence of pathological tau within brain cells and cognitive impairments evident in virtually all tauopathies, including AD.^22^,^31^,^34^ Efficient axonal transport is essential for the delivery of newly synthesized proteins, lipids, and organelles to synapses, and for the removal of damaged components. The defective axonal transport mechanisms in AD patients could be observed due to synaptic dysfunction, axonal swelling, and neuronal death. The dysregulation of ribosome biogenesis and protein synthesis, association of pathological tau proteins with ribosomes impairs protein synthesis, reduces the production of proteins vital for synaptic plasticity may potentially lead to cognitive decline in AD patients. Dysregulated protein synthesis affects cellular stress responses and proteostasis, which adversely affects neuronal health.^33^-^36^

### Strength of the Study:

The main strength of our study is that we generated a comprehensive NGKD analysis of RNA-seq data from DLPFC tissues from patients with severe AD, and our approach is highly effective for analyzing high-throughput expression data to decode differentially regulated molecular pathways related to severe AD. In addition, our study is particularly helpful for decoding complex diseases, such as AD, in which the interaction of an array of pathological mechanisms is responsible for the entire disease state. The key DEGs deduced from our study may be further explored in detail using appropriate in vitro and in vivo systems and the latest artificial intelligence/machine learning classification algorithms to identify biomarker signatures associated with the diagnosis, staging, or monitoring of AD progression.

### Limitations

However, our study has some limitations due to the small number of samples in the AD group and reliance on only high-dimensional datasets derived from RNA sequencing. Hence, the identification of molecular pathways using RNA-seq and other “omics” datasets and extensive NGKD analysis is essential to develop and design experimental strategies for personalized treatment regimens for patients with AD.

## CONCLUSIONS

Our study unraveled the complex molecular pathways involved in AD pathogenesis. This includes the disruption of energy metabolism, protein synthesis, axonal transport, and immunological responses. Importantly, we obtained additional insights into the cascade of molecular pathways leading to severe AD and identified important avenues for therapeutic intervention. Our findings require further validation using appropriate model systems and should concentrate mainly on their potential for developing novel diagnostic tools and therapeutic strategies for AD. In the future, the functional effects of the dysregulated pathways identified in this study should be justified using appropriate *in vitro* models, such as induced pluripotent stem cell (iPSC)-derived neurons or glial co-cultures, and *in vivo* AD models. As stated in our limitations, these analyses should be performed in larger and more diverse AD patient cohorts. Finally, integrating proteomics, glycomics, metabolomics, and epigenomics is necessary to construct a more complete model of AD and to fully understand the sequence of events that lead from molecular dysregulation to severe cognitive decline. In conclusion, our findings add to the existing knowledge of the pathophysiology of severe AD and will help in the development of more effective strategies for diagnosis, therapy, and prevention.

### Authors’ contribution:

**HK & PNP** conceived, designed, writing original draft, review and editing; are responsible for integrity of research.

**HK, AA, MIN & PNP** did data collection, data curation, data analysis and have read and agreed to the final version of the manuscript.
